# Corrigendum: Hydrogen sulfide reduces oxidative stress in Huntington’s disease via Nrf2

**DOI:** 10.4103/NRR.NRRONLINE-D-26-00303

**Published:** 2026-04-28

In the article titled "Hydrogen sulfide reduces oxidative stress in Huntington’s disease via Nrf2," published in *Neural Regeneration Research* (Jiang et al., 2025), the Y-axis unit in Figure 2F was incorrectly labeled as it was directly output from the behavioral system software. The authors have recalculated the data and the corrected Figure 2F is shown below.


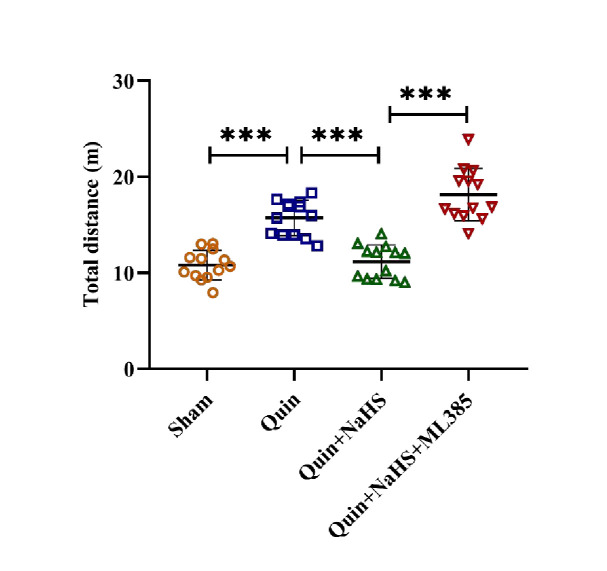


All subsequent interpretations and conclusions in the article remain valid and are supported by the corrected figure.

The online version of the original article can be found at DOI: 10.4103/NRR.NRR-D-23-01051.
